# 3D-Printed Multi-Axis Alignment Airgap Dielectric Layer for Flexible Capacitive Pressure Sensor

**DOI:** 10.3390/mi15111347

**Published:** 2024-10-31

**Authors:** Jeong-Beom Ko, Soo-Wan Kim, Hyeon-Beom Kim, Hyeon-Yun Jeong, Su-Yeong Moon, Young-Jin Yang

**Affiliations:** Clean Energy Transition Group, Korea Institute of Industrial Technology (KITECH), Jeju 63243, Republic of Korea; no1kori@kitech.re.kr (J.-B.K.); swkim89@kitech.re.kr (S.-W.K.); beom0406@kitech.re.kr (H.-B.K.); yoon9475@kitech.re.kr (H.-Y.J.); tndud194@kitech.re.kr (S.-Y.M.)

**Keywords:** capacitive pressure sensor, 3D printing, alignment airgap dielectric, flexible sensor

## Abstract

Flexible pressure sensors are increasingly recognized for their potential use in wearable electronic devices, attributed to their sensitivity and broad pressure response range. Introducing surface microstructures can notably enhance sensitivity; however, the pressure response range remains constrained by the limited volume of the compressible structure. To overcome this limitation, this study implements an aligned airgap structure fabricated using 3D printing technology. This structure, designed with a precisely aligned triaxial airgap configuration, offers high deformability under pressure, substantially broadening the pressure response range and improving sensitivity. This study analyzes the key structural parameters—the number of axes and pore size—that influence the compressibility and stability of the dielectric material. The results indicate that the capacitive pressure sensor with an aligned airgap structure, manufactured via 3D printing, exhibits a wide operating pressure range (50 Pa to 500 kPa), rapid response time (100 ms), wide limit of detection (50 Pa), and approximately 21 times enhancement in sensitivity (~0.019 kPa^−1^ within 100 kPa) compared with conventional bulk structures. Furthermore, foot pressure monitoring trials for wearable sensor applications demonstrated exceptional performance, indicating the sensor’s suitability as a wearable device for detecting plantar pressure. These findings advocate for the potential of 3D printing technology to supplant traditional sensor manufacturing processes.

## 1. Introduction

Flexible pressure sensors, capable of detecting parameters such as force, vibration, and noise, are increasingly sought after for applications in wearable electronic devices [1,2,3], human–machine interaction systems [4,5,6], and intelligent robots [7]. Traditional sensor fabrication techniques, such as photolithography, are limited by their inflexibility, increased procedural steps, and the high costs associated with device production. In response, there have been proposals for new fabrication methods [8,9,10]. Among these, 3D printing technology stands out as a promising approach to the production of flexible pressure sensors [11]. This method enables the simple and cost-effective creation of structures with complex shapes and features, positioning it as a critical player in developing flexible pressure sensors [12,13].

Flexible pressure sensors generate electrical signals when subjected to external pressure, operating through mechanisms that convert these signals [14,15]. Typically, there are three detection principles: piezoresistive [16,17], piezoelectric [18,19], and capacitive [20,21,22]. Each principle offers unique advantages and drawbacks. Piezoresistive sensors, which utilize the ability of piezoresistive materials to change resistance under stress, are known for their high output voltage and sensitivity but are prone to environmental variability. Piezoelectric sensors, noted for their high sensitivity and minimal electromagnetic interference, face challenges in calibration and are primarily suitable for dynamic measurements.

Capacitive sensors, which measure changes in capacitance due to the movement of electrodes separated by a dielectric layer, exhibit high sensitivity and stability against external environmental factors. However, they often suffer from nonlinearity and a limited pressure range due to the low compressive strain and high Young’s modulus of the dielectric polymer layer. Despite these challenges, capacitive pressure sensors continue to be explored using various materials, designs, and strategies to enhance their pressure detection range and improve sensitivity [23,24]. Previous studies have demonstrated that incorporating microstructures such as pillars [25], pyramids [26], convex shapes [27], and waves [28] within the dielectric layer of flexible pressure sensors effectively enhances sensitivity. However, fabricating microstructure silicon molds incurs high costs, extensive time, and complex procedural steps. Various alternative methods, including the particle templating method [29], the chemical foaming method [30], and the emulsion templating method [31], have been proposed to form porous structures within the dielectric layer. Nonetheless, conventional methods often yield irregular internal pore structures, complicating the achievement of uniform performance across sensors produced in the same batch. Additionally, these irregular microstructures tend to compromise the durability of pressure sensors from a mass production perspective. Consequently, sensor structures that concurrently enhance sensitivity, linearity, and the detection range need to be developed.

This study introduces a novel approach to fabricating capacitive pressure sensors using a three-axis alignment airgap structure, employing 3D printing technology. The sensor comprises a 3D-printed photocurable polymer dielectric layer sandwiched between upper and lower electrodes. The fabrication process avoids complex steps in creating the dielectric layer. The sensor performance was significantly improved without adding functional materials by optimizing the number of axes and pore sizes within the pure dielectric alignment airgap structure. The geometric structures and detection mechanisms of the fabricated aligned airgap capacitive pressure sensor were systematically analyzed. Additionally, the sensor was integrated into a shoe insole to monitor the plantar pressure of the human body. This study confirms the applicability of 3D-printed capacitive pressure sensors and highlights their potential use in wearable devices for human health monitoring, particularly in medical and sports injury detection contexts.

## 2. Experimental

**Figure 1:**
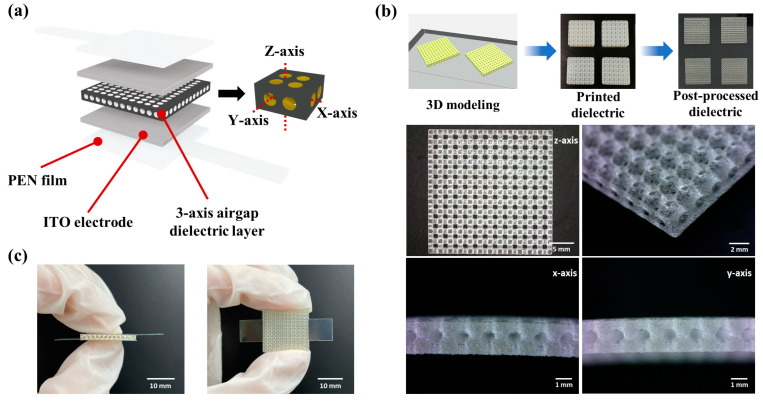
Fabrication process and structure of the device. (**a**) Schematic diagram of the flexible ITO PEN film electrodes (**top** and **bottom**) and 3-axis alignment airgap dielectric layer (**middle**). (**b**) Manufacturing process and results for 3-axis alignment airgap structured dielectrics. (**c**) Photograph of the capacitive pressure sensor.

The 3-axis alignment airgap pressure sensor was designed as depicted in Figure 1a. To optimize the sensitivity and suitability of wearable devices, parameters such as the number of axes and pore sizes of the airgap were defined, and the device was fabricated with dimensions of 20 mm (W) × 20 mm (D) × 2 mm (H) (Figure S1). The fabrication process and results for the 3-axis alignment airgap dielectric layer are illustrated in Figure 1b. This process employed a PolyJet 3D printer (J850, Stratasys, Edina, MN, USA). The dielectric layer was constructed using a flexible polymer, Agilus30 (FLX935, Stratasys, Edina, MN, USA), and a support material (SUP706B, Stratasys, Edina, MN, USA). Post-printing, the dielectric layer underwent treatment in an alkaline solution consisting of 2% sodium hydroxide in DI-water, followed by the removal of the support material using a post-processing washing system (SCA3600, Stratasys, Edina, MN, USA). To eliminate residual moisture and ensure the complete curing of the dielectric layer, it was exposed to UV light for 1 h. The electrodes of the pressure sensor were fabricated using a 125 μm thick polyethylene naphthalate (PEN) film coated with a 180 nm thick indium tin oxide (ITO) layer (MTI, Richmond, CA, USA). This structure incorporated the alignment airgap dielectric layer between the film electrodes to assemble the flexible sensor. A photograph of the fabricated pressure sensor with the three-axis alignment airgap is shown in Figure 1c. A network analyzer (E5061B, Keysight, Colorado Springs, CO, USA) was employed to analyze the dielectric constant of the material. The load control of the pressure sensor was managed using a tension/compression stand (ESM303, Mark-10, Copiague, NY, USA) and a force gauge with a 0.5 N resolution (M5-100, Mark-10, USA). The sensor’s capacitance was evaluated using a semiconductor device analyzer (B1500A, Keysight, Colorado Springs, CO, USA) (Figure S2).

## 3. Results and Discussion

**Figure 2:**
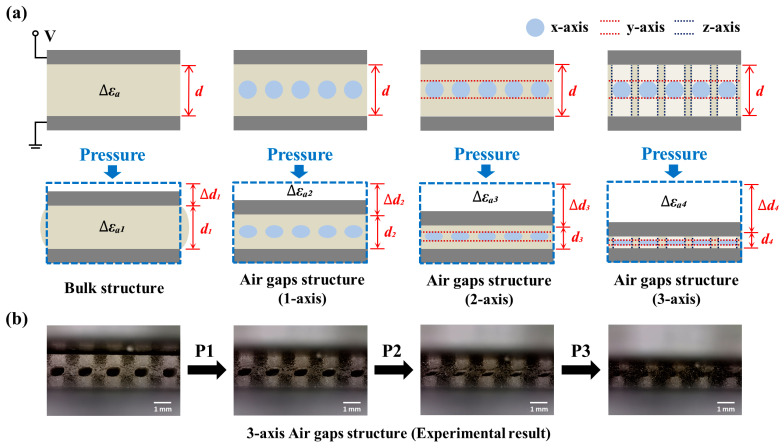
Schematic of the mechanism of the capacitive sensor using a multi-axis alignment airgap dielectric layer. (**a**) Change in electrode height displacement according to the number of axes under an identical pressure. (**b**) Result of height displacement variation in dielectric layer with pressure change.

The capacitive pressure sensor, designed with a parallel-plate capacitor structure, comprises two parallel electrodes and an elastic dielectric layer positioned between them. The capacitance of this sensor changes in response to variations in three key parameters: the area of the electrodes (A), the relative dielectric constant of the dielectric material $\left(\varepsilon_{r}\right)$, and the distance between the electrodes (d).

(1) $$C=\frac{A\varepsilon_{r}\varepsilon_{0}}{d}$$

where $\varepsilon_{0}$ is the permittivity of air, and $\varepsilon_{r}$ is the permittivity of the dielectric layer. In capacitive sensors with a porous dielectric layer, the capacitance changes as applied pressure causes the pores to close, replacing the permittivity of air inside the capacitor with that of the dielectric material. This alteration in the overall capacitance results from the increase in the dielectric constant of the layer. This study introduces a 3-axis alignment airgap structure for the dielectric layer, which cannot be achieved using conventional mold fabrication techniques; thus, 3D printing technology is used instead. This innovation results in a flexible capacitive sensor with a broad detection range and enhanced sensitivity. Figure 2a illustrates the schematic changes in $\varepsilon_{r}$ and d when the same pressure is applied to four distinct sensor structures. Figure 2b shows the height displacement variation in the dielectric layer in a 3-axis alignment airgap structure (900 μm pore size) under different pressure conditions (P1 < P2 < P3). The results demonstrate decreased dielectric layer height displacement with increasing pressure.

**Figure 3:**
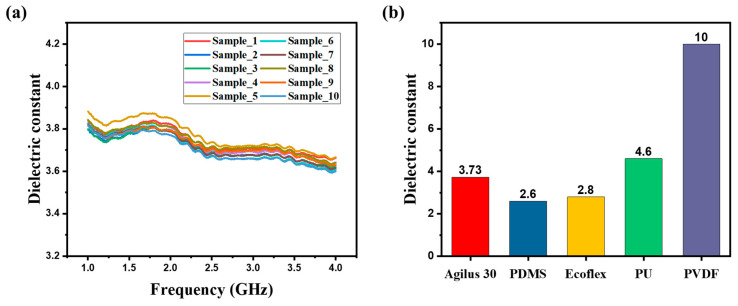
Characterization of the dielectric constant measured at 1–4 GHz. (**a**) Dielectric constant for 10 samples. (**b**) Bar charts comparing the dielectric constant reported in the literature using dielectric materials.

When the dielectric layer deforms under pressure, the low dielectric constant of air ($\varepsilon_{air}=1)$ is substituted by the higher dielectric constant of the dielectric material. The effective dielectric constant of the airgap dielectric is calculated by combining the dielectric constant of air with that of the remaining dielectric layer, proportional to the volume occupied by the air layers.

(2) $$\varepsilon_{r}=\varepsilon_{air}V_{air}+\varepsilon_{p}V_{p}$$

where $\varepsilon_{air}$ is the dielectric constant of air, $V_{air}$ is the volume fraction of air, $\varepsilon_{p}$ is the dielectric constant of the dielectric, and $V_{p}$ is the volume fraction of the dielectric. As the dielectric layer is compressed by the applied pressure, the air is continuously expelled, and $V_{p}$ increases. At the same time, $V_{air}$ decreases, resulting in an increase in the effective dielectric constant of the dielectric layer. To assess the suitability of using the flexible polymer Agilus 30 as the dielectric material in capacitive pressure sensors, specimens were produced with an inner diameter of 3.0 mm and an outer diameter of 7.0 mm. These specimens were evaluated using a network analyzer (Figure S3). The graph in Figure 3a displays the dielectric constant results for these specimens. The measurement of the dielectric constant was repeated for 10 specimens at a frequency range of 1–4 GHz, and the average dielectric constant for each specimen was measured. The measurement results show an average dielectric constant of approximately 3.73 at a 1–4 GHz frequency range. Figure 3b presents a comparison of the dielectric constant of Agilus 30 with commonly used materials such as polydimethylsiloxane (PDMS) [32] and Ecoflex [33], demonstrating that Agilus 30 has about 1.3 times the dielectric constant. Thus, the flexible polymer Agilus 30 exhibits substantial potential for use as a dielectric material in capacitive pressure sensors.

Further validation of the structural feasibility and optimization of the capacitive sensor was conducted through finite element structural simulations using ANSYS software(ANSYS 2021 R2, Canonsburg, PA, USA). These simulations examined the distance between electrodes in relation to the dielectric’s number of axes and pore sizes under a consistent pressure of 35 kPa (Figure S4). The analysis verified that the height displacement increased with the number of axes, from the bulk form to the three-axis structure. Specifically, the structure with three axes and a 900 μm pore size exhibited an approximately 15 times greater height displacement compared to the bulk structure under the same pressure. This finding indicates that the pressure sensor with the three-axis alignment airgap structure is more compressible, thereby enhancing sensitivity due to significant changes in the dielectric constant and electrode displacement under compression.

The volume fraction of the air and dielectric material was calculated based on the measured dielectric constant and volume values for each structure according to the airgap configuration. The initial effective dielectric constant was then compared across different structures. It was observed that a lower initial effective dielectric constant leads to a greater change in the effective dielectric constant when pressure is applied. As shown in Table 1, as the number of axes increases and the pore size becomes larger, the effective dielectric constant decreases, which enhances the sensor performance.

Through simulation and a dielectric constant analysis of the airgap structural model, it was confirmed that the number of axes and pore size affect sensor performance. The highest sensor performance was observed with a three axes and 900 μm pore size. This configuration showed significant height displacement and a low initial dielectric constant.

The sensitivity of the pressure sensor (S) was calculated based on the relative change in capacitance resulting from the applied external pressure. The sensitivity of the pressure sensor is defined as the ratio of the relative change in capacitance to the change in the applied external pressure, expressed mathematically as follows:

(3) $$S=\delta \frac{∆C}{C_{0}}/\delta P,$$

where ∆C is the change in the capacitance of the sensor due to the applied pressure, $C_{0}$ is the initial capacitance of the sensor without external pressure, and P is the external pressure applied to the sensor.

**Figure 4:**
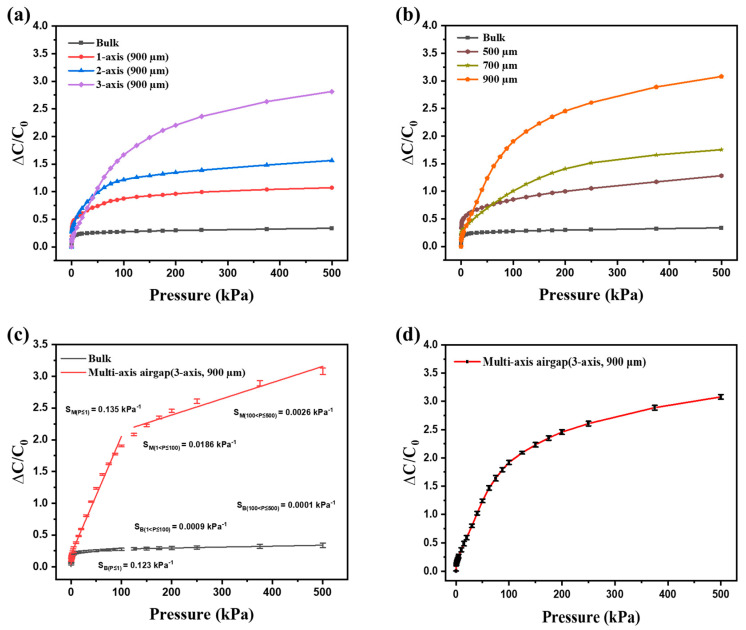
Pressure response curves of sensors for various structures (**a**) with 1–3 axes. (**b**) Pore size 500–900 μm. (**c**) Comparison of the sensor’s performance, alignment airgap and bulk. (**d**) Relative capacitance curves for the 20 sensor samples.

We analyzed the relative capacitance and pressure variation in the range of 1–500 kPa for a number of axes and pore sizes in the alignment airgap pressure sensor. Figure 4a illustrates the relative capacitance based on the number of axes. The highest relative capacitance was observed in the three-axis configuration. Notably, the dielectric layer of the three-axis alignment airgap structure incorporates more airgaps compared to the bulk, one-axis and two-axis configurations, inducing a more significant change in the height displacement between the electrodes under identical pressure conditions. As the pore size decreases, the sensor performance improves in the low-pressure range (P < 1 kPa), but the overall performance across the entire pressure range decreases, as shown in Figure 4b. The fabricated sensor demonstrates that its sensitivity decreases as pressure increases. The pressure range is divided into P > 1 kPa, 1 kPa < P < 100 kPa, and 100 kPa < P < 500 kPa. The relative capacitance and sensitivity for each pressure range are shown in Figures S5 and S6.

The sensor with a 3-axis configuration and 900 μm pore size demonstrated the highest response, showing that sensor performance varies with the parameters of the alignment airgap structure. To evaluate the response and sensitivity of the fabricated pressure sensor, the performance of the three-axis and 900 μm structure with the highest response was compared to that of a conventional bulk structure, as shown in Figure 4c. Pressure points and ranges were specifically chosen based on the measurement requirements for a low-pressure range (P ≤ 1 kPa), mid-pressure range (1 kPa ≤ P ≤ 100 kPa), and high-pressure range (100 kPa ≤ P ≤ 500 kPa). The sensitivity of the three-axis and 900 μm structure improved up to 21 times compared to the bulk structure samples, with sensitivities recorded at 0.135 kPa⁻^1^, 0.0186 kPa⁻^1^, and 0.0026 kPa⁻^1^, respectively. Figure 4d shows that to ensure the selected structure’s performance consistency, 20 pressure sensors with the same three-axis and 900 μm configuration were 3D printed, and the capacitance changes were analyzed. The measurement results of the 20 sensors’ averages and standard errors demonstrated that the standard error values were less than 0.05 in all regions, indicating high performance consistency across all the sensors fabricated using 3D printing. These findings affirm that the proposed method can cover a relatively wide range of pressure, and can achieve the reproducibility and repeatability of the sensor due to the control of the aligned air gap distribution through 3D printing. Furthermore, the performance was compared with existing capacitive pressure sensors based on porous/airgap dielectrics, as shown in Table S1 [34,35,36,37,38,39].

**Figure 5:**
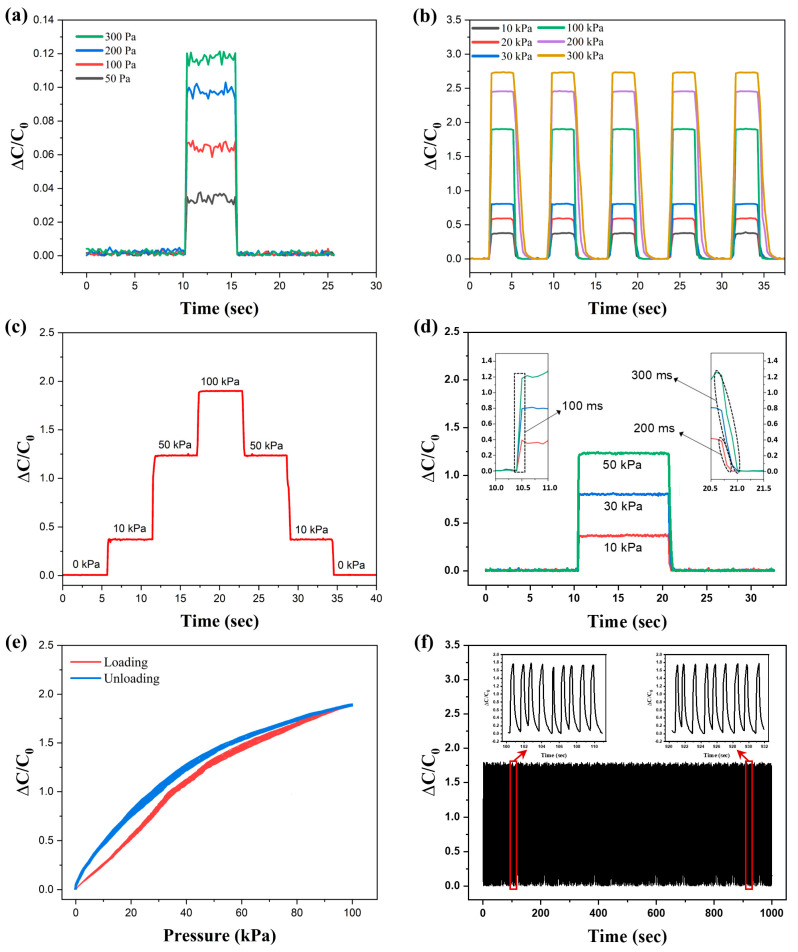
Characterization of the pressure sensing performance of the three-axis alignment airgap pressure sensor. (**a**) Limit of detection. (**b**) Relative capacitance change when cycling the sensor through loading/unloading at different pressures. (**c**) Relative capacitance variation at different external pressures. (**d**) Response/recovery times of the sensor at 10, 30, and 50 kPa. (**e**) Hysteresis curves of loading/unloading pressure. (**f**) Durability test of the sensor for 1000 cycles.

In addition to sensitivity, essential performance indicators for sensors encompass the detection limit, response time, stability, and repeatability. For a more comprehensive examination of sensor performance, a capacitive sensor based on the three-axis and 900 μm structure was selected. As shown in Figure 5a, the fabricated sensor is capable of detecting subtle pressures as low as 50 Pa. Figure 5b demonstrates the sensor’s response to cyclic pressures of 10 kPa, 20 kPa, 30 kPa, 100 kPa, 200 kPa, and 300 kPa. Across five cycles, the sensor produced almost identical outputs at each pressure level, increasing capacitance with rising pressure. This consistency indicates the sensor’s ability to differentiate between various pressure levels accurately. Despite an observed increase in the dielectric recovery time during the unloading of pressures above 200 kPa, the sensor remains suitably applicable for wearable devices that operate within pressure ranges below 100 kPa. Stable capacitance was also noted under stepwise pressure inputs of 10 kPa, 50 kPa, and 100 kPa, as depicted in Figure 5c. The sensor’s response to/recovery time for pressure changes are shown in Figure 5d, where the capacitance rapidly increased within 100 ms with loading pressures of 10 kPa, 30 kPa, and 50 kPa and maintained stability. Upon unloading, the capacitance returned to its initial state within 300 ms. A hysteresis analysis of the pressure sensor is presented in Figure 5e, with a hysteresis error of 8.6%, reflecting a commendable performance. Lastly, Figure 5f displays the results of a 1000 cycle repeatability test under a 100 kPa load, where the capacitance variation remained nearly identical, demonstrating exceptional stability and durability. These results affirm the superior characteristics of the detection limit, detection range, linearity, and stability of the fabricated three-axis alignment airgap pressure sensor, highlighting its practical value for pressure detection in real-world applications.

**Figure 6:**
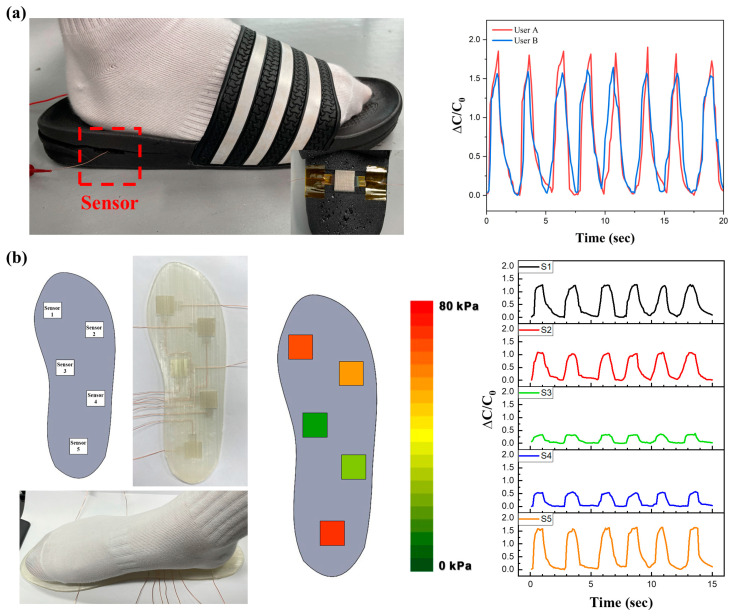
Practical applications of the three-axis alignment airgap pressure sensor. (**a**) Capacitive sensor attached to the slipper (**left**), and relative capacitance variation in the planter pressure for user A and B (**right**). (**b**) Five capacitive sensors attached to the 3D-printed shoe insole (**left**), and the corresponding force distribution on the five sensors when foot stepping (**right**).

The three-axis alignment airgap capacitive pressure sensor, characterized by its high sensitivity, rapid response, and cyclic stability, is well-suited for wearable devices that monitor human movements in real time by detecting physical signals from the body. Among the application areas of pressure sensors, measuring the pressure distribution of the planter is performed in various fields, and analyzing gait patterns can help confirm balance or judge rehabilitation treatment and structural problems in the foot. Therefore, the fabricated sensors can be integrated into the insole to collect various gait patterns. As depicted in Figure 6a, the sensor was inserted into the heel of a commercial EVA (Ethylene Vinyl Acetate) slipper to monitor the walking motion of users weighing 50 kg and 70 kg for 20 s. During each step, the capacitance value significantly increased and swiftly returned to its original state as the foot was lifted from the ground. This change in capacitance effectively captures the difference in output values corresponding to the user’s weight. Building on these observations, five pressure sensors were designed and 3D printed for integration into an insole, strategically positioned at various locations to map the plantar pressure distribution, as illustrated in Figure 6b. These sensors were installed in the fabricated insole, and the plantar pressure distribution was measured as a 50 kg user stepped in place. It was observed that the pressure on each sensor increased, leading to a rise in capacitance when the user stepped. The pressure distribution across sensors S1–S5 was measured as 26.3%, 22.4%, 6.7%, 11.5%, and 33.1%, respectively, illustrating how the user’s weight was distributed across the foot. These findings affirm that the pressure sensors, employing 3D printing technology, exhibit exceptional stability and detection capabilities in real-world scenarios, validating their practicality in applications related to plantar pressure measurement.

## 4. Conclusions

This study introduced a novel method for fabricating capacitive pressure sensors with high sensitivity and a broad detection range using PolyJet 3D-printing technology to create a flexible dielectric layer. The fabrication process, simplified through 3D printing, streamlined the production of these sensors. The dielectric layer of the sensor features a three-axis alignment airgap structure designed to deform under minimal pressure, ensuring outstanding sensor performance. Experimental evaluations revealed that the fabricated three-axis alignment airgap capacitive sensor achieved a sensitivity of 0.0186 kPa⁻^1^, limit of detection of 50 Pa, a rapid response time of less than 300 ms, and stable performance over more than 1000 loading/unloading cycles. Additionally, it demonstrated up to 21 times higher sensitivity than bulk-type capacitive sensors using the same material. The sensor was successfully employed to monitor human motion, specifically plantar pressure, and accurately detected the signals generated by users of varying weights during movement. It was also proven to effectively provide plantar pressure distribution when integrated with 3D-printed insoles and sensors. Consequently, this 3D-printed structure-controlled pressure sensor shows significant potential for future applications in human–computer interfaces, medical assistive devices, and wearable technologies, highlighting its versatility and effectiveness in diverse practical scenarios.

## Figures and Tables

**Table 1:** Effective dielectric constant and volume based on the three-axis alignment airgap structure in the initial state.

	Pore Size: 900 μm				Number of Axes: 3		
Type	Bulk	1-Axis	2-Axis	3-Axis	500 μm	700 μm	900 μm
Dielectric Volume (mm^3^)	800	647.3	564.6	457.6	675	574.1	457.6
Dielectric constant	3.73	3.18	2.87	2.47	4.28	2.9	2.47

## Data Availability

The original contributions presented in the study are included in the article/Supplementary Materials, further inquiries can be directed to the corresponding author.

## Supplementary Materials

The following supporting information can be downloaded at: https://www.mdpi.com/article/10.3390/mi15111347/s1, Figure S1: 3D modeling diagram of multi-axis alignment airgap structure dielectric layer for number of axis and pore size; Figure S2: Schematic of the 3-axis alignment airgap capacitive pressure sensor testing set-up; Figure S3: Dielectric constant measurement equipment and specimen; Figure S4: Simulation of the capacitive pressure sensor and height displacement results for compression; Figure S5: Change in relative capacitance of pressure sensor for applied pressure range (0–1 kPa, 1–100 kPa, 100–500 kPa); Figure S6 Sensitivity of capacitive pressure sensors for various structures (number of axis, pore size) and applied pressure ranges from 0–500 kPa; Table S1: Performance comparison of the three-axis alignment airgap pressure sensor fabricated in this research and precedent research.
