# Supplementary material for: 3D-Printed Multi-Axis Alignment Airgap Dielectric Layer for Flexible Capacitive Pressure Sensor
Source: Micromachines (Basel). 2024 Oct 31;15(11):1347. doi: 10.3390/mi15111347 (PMC11596244; doi:10.3390/mi15111347)
Supplement: Supplementary file 1 [file micromachines-15-01347-s001.zip › micromachines-3278455-supplementary.pdf]

Supplementary Materials for

3D-Printed Multi-Axis Alignment Airgap Dielectric Layer for Flexible Capacitive Pressure Sensor

Jeong Beom Ko[1], Soo Wan Kim [1], Hyeon Beom Kim[1], Hyeon Yun Jeong[1], Su Young Moon[1] and Young Jin Yang [1,*]

[1] Clean Energy Transition Group, Korea Institute of Industrial Technology (KITECH), Jeju 63243, Korea

This PDF file includes:

Figure S1, Figure S2, Figure S3, Figure S4, Figure S5 and Table S1

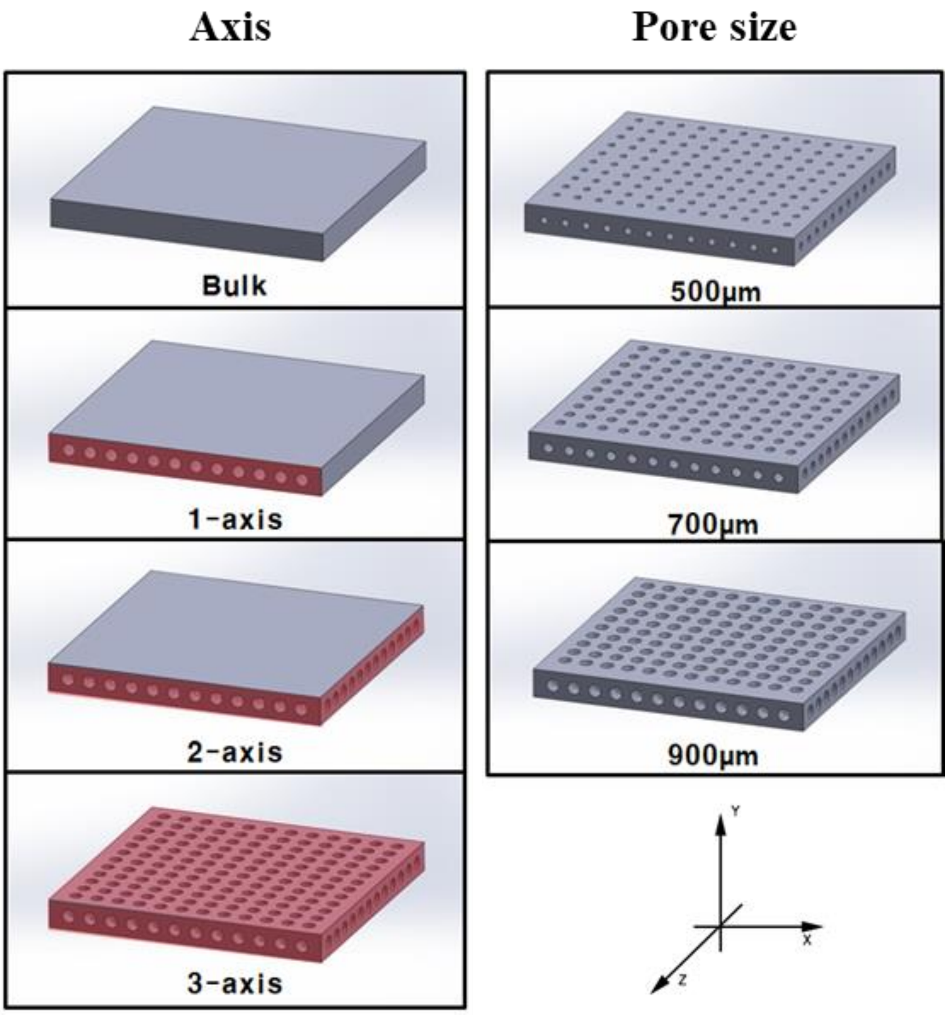

Figure S1. 3D modeling diagram of multi-axis alignment airgap structure dielectric layer for number of axis and pore size

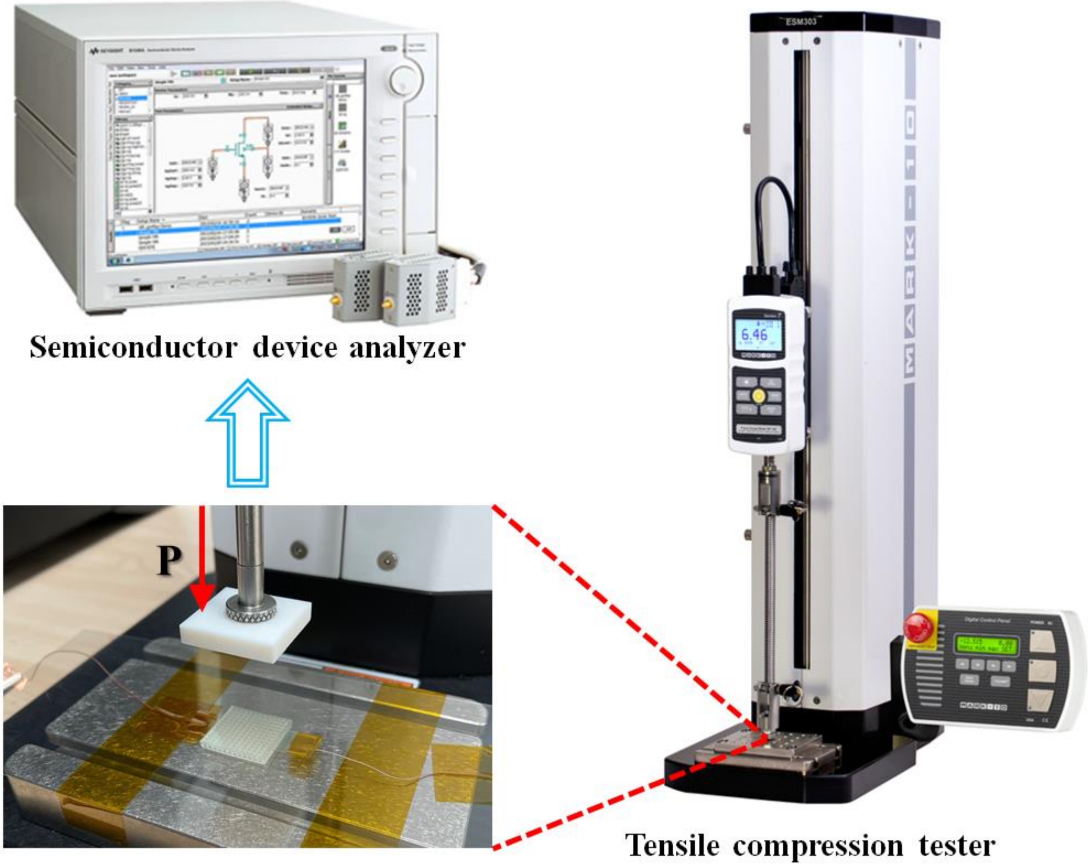

Figure S2. Schematic of the 3-axis alignment airgap capacitive pressure sensor testing set-up.

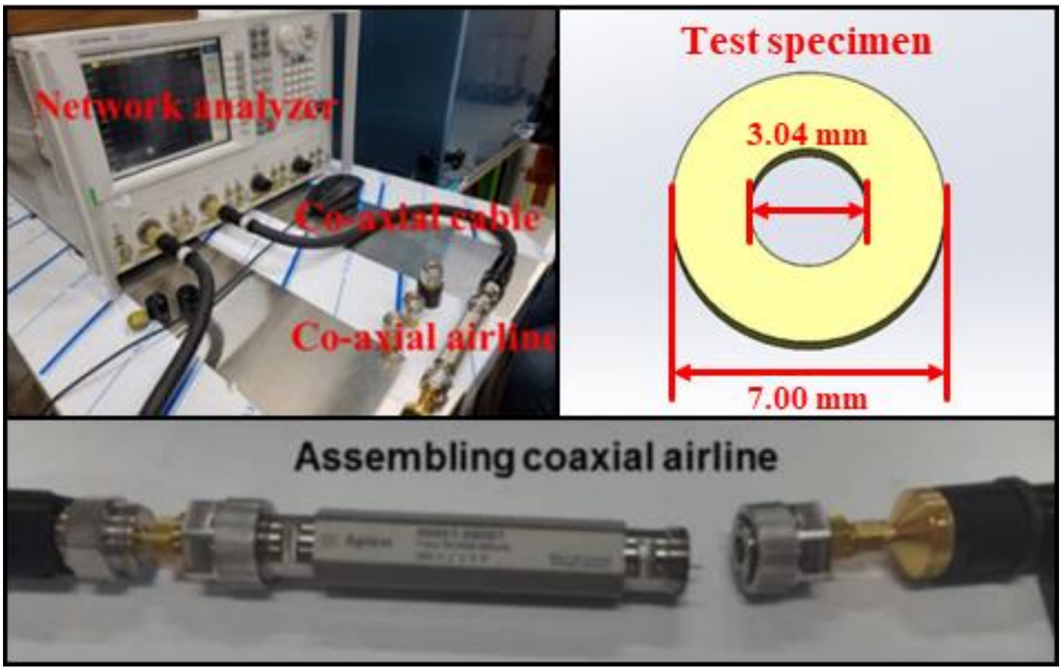

Figure S3. Dielectric constant measurement equipment and specimen.

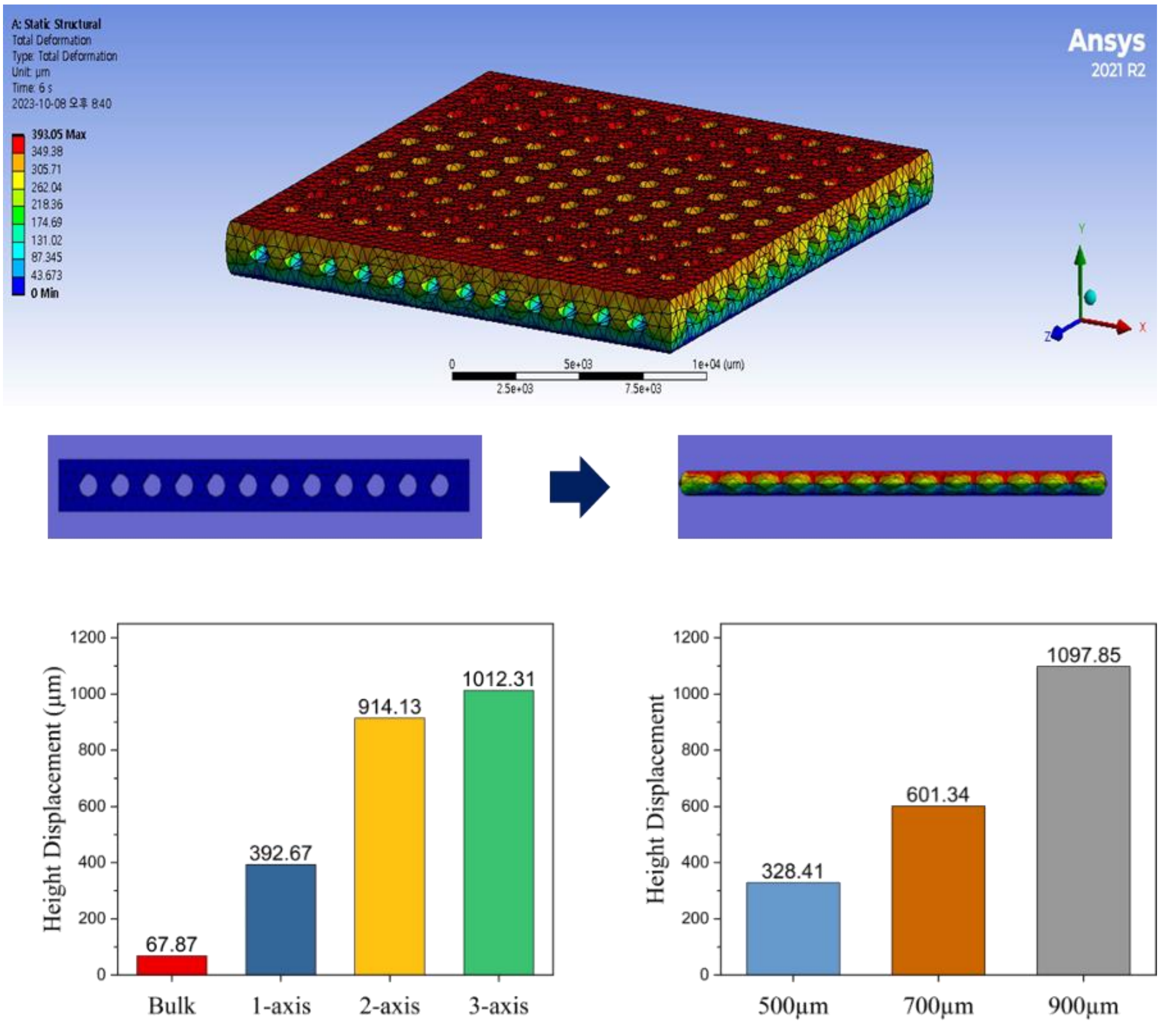

Figure S4. Simulation of the capacitive pressure sensor and height displacement results for compression.

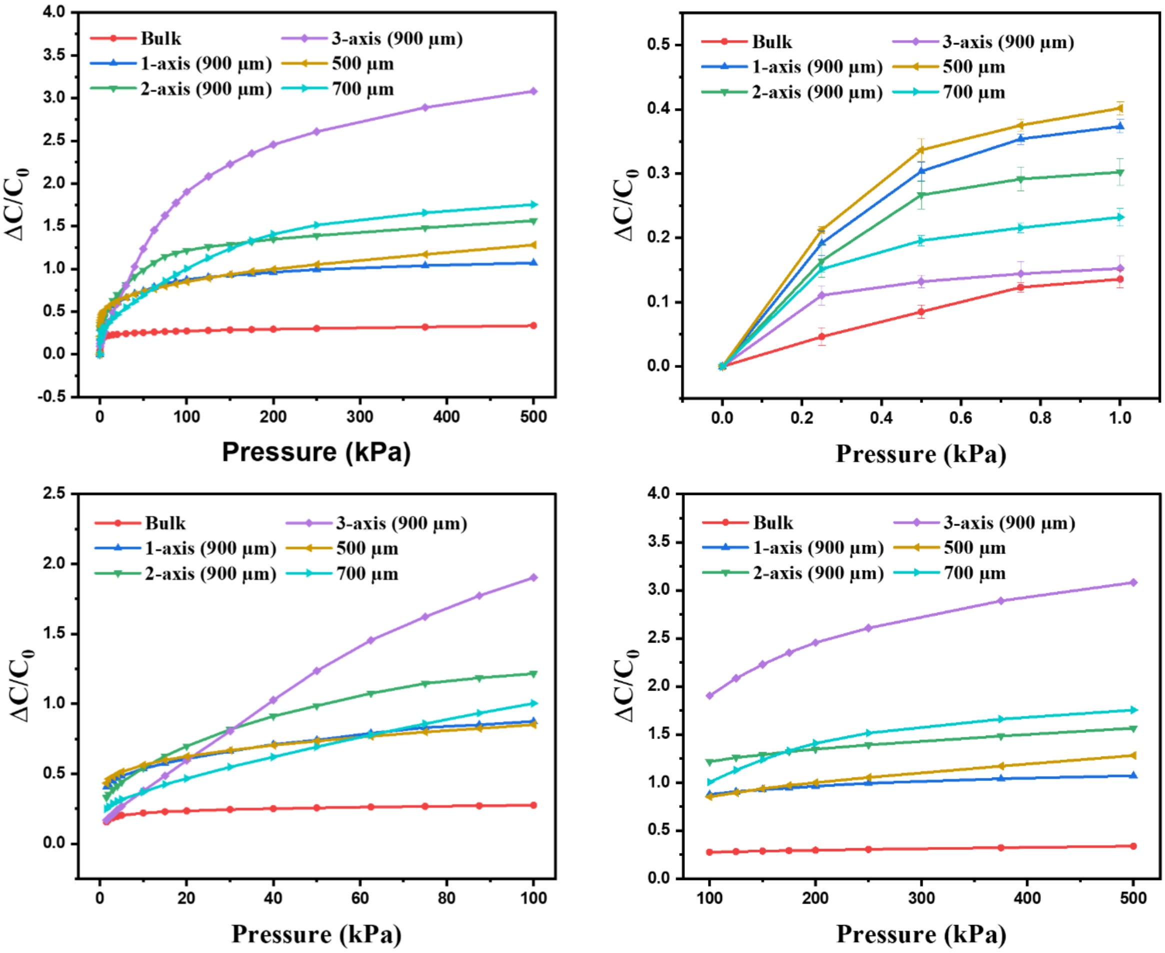

Figure S5. Change in relative capacitance of pressure sensor for applied pressure range (0-1 kPa, 1-100 kPa, 100-500 kPa)

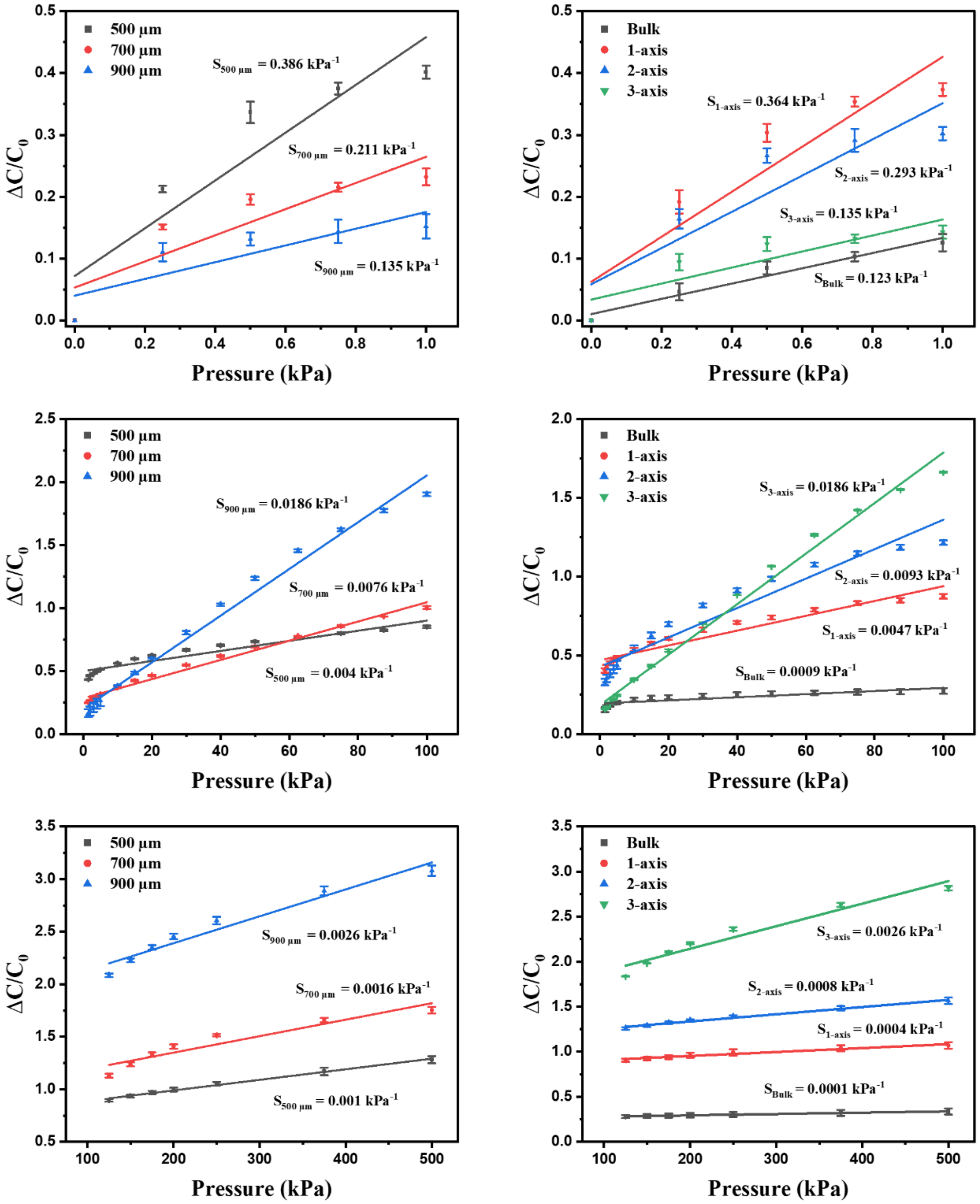

Figure S6. Sensitivity of capacitive pressure sensors for various structures(number of axis, pore size) and applied pressure ranges from 0-500 kPa.

Table S1. Performance comparison of the 3-axis alignment airgap pressure sensor fabricated in this research and precedent research.

Material/Structure	Sensitivity (Pressure range)	Pressure range (kPa)	consistency of performance	Reference
Micropillar	0.42 kPa^{-1}(≤ 1.5 kPa)	50	O	[34]
Microdome	0.0044 kPa^{-1}(≤ 11 kPa)	500	O	[35]
Micropyramid	0.55 kPa^{-1}(≤ 0.2 kPa)	8	O	[36]
Porous (emulsion template method)	1.18 kPa^{-1}(≤ 0.02 kPa)	5	X	[37]
Porous (particle template method)	0.51 kPa^{-1}(≤ 10 kPa)	500	X	[38]
Porous (chemical foaming method)	0.0052 kPa^{-1}(≤ 20 kPa)	900	X	[39]
3-axis Alignment Airgap	0.135 kPa^{-1}(≤ 1 kPa)<br>0.0186 kPa^{-1}(≤ 100 kPa)<br>0.0026 kPa^{-1}(≤ 500 kPa)	500	O	This work
